# Data regarding clinical evaluation of collagen membrane in oral mucosal defects due to oral submucous fibrosis and leukoplakia

**DOI:** 10.1016/j.dib.2018.03.070

**Published:** 2018-03-22

**Authors:** Veeraiah Chowdary Jasthi, Thorakkal Shamim

**Affiliations:** a Department of Oral and Maxillofacial Surgery, Anil Neerukonda Institute Of Dental Science, Visakhapatnam, India; b Department of Dentistry, Government Taluk Head Quarters Hospital, Malappuram, India

## Abstract

This data depicts the clinical evaluation of collagen membrane in oral mucosal defects due to oral submucous fibrosis and leukoplakia. This data comprised of 10 patients in the age group of 20–60 (3 male and 2 female patients with grade III and grade IV oral submucous fibrosis and 5 male patients with oral leukoplakia with a size of 3–5 cm in diameter). The parameters such as pain, swelling, allergy, biodegradability of collagen membrane, degree of re-epithelisation, degree of contracture, mouth opening and wound size were assessed after the placement of collagen membrane in oral mucosal defects. There is less data regarding the usage of collagen membrane as a biological dressing material to cover mucosal defects (Rastogi et al., 2009) [1].

## Specifications Table

**Table:** 

Subject area	*Medicine and Dentistry (Oncology)*
More specific subject area	*Oral Potentially malignant lesions (oral submucous fibrosis and leukoplakia)*
Type of data	*Table, figure*
How data was acquired	*Clinical and histopathological analysis*
Data format	*Clinical data*
Experimental factors	*Parameters such as pain, swelling, allergy, biodegradability of collagen membrane, degree of re-epithelisation, degree of contracture, mouth opening and wound size after placement of collagen membrane in oral mucosal defects*
Experimental features	*Clinical evaluation of collagen membrane in oral mucosal defects due to oral submucous fibrosis and leukoplakia*
Data source location	*Anil Neerukonda Institute Of Dental Science, Vishakapatanam, India.*
Data accessibility	*Data are included in the paper*

## Value of the data

• These data describe the clinical evaluation of collagen membrane in oral mucosal defects due to oral submucous fibrosis and leukoplakia.
• These data depicts pictorial information about clinical evaluation of oral potentially malignant lesions (oral submucous fibrosis and leukoplakia).
• These data depicts that collagen membrane can be advocated for use in the mouth to cover large areas devoid of mucous membrane.
• These data describe improvement of mouth opening ranged from 32 to 44 mm in cases of oral submucous fibrosis.
• The data with larger sample size with longer follow up is mandatory to authenticate this research and it can be carried out with collaborative effort.

## 1 Data

.

## 2 Experimental design and materials and methods

There is less data regarding the usage of collagen membrane as a biological dressing material to cover mucosal defects [1] This data depicts the clinical evaluation of collagen membrane in oral mucosal defects due to oral submucous fibrosis and leukoplakia. This data comprised of 10 patients in the age group of 20–60 (3 male and 2 female patients with grade III and grade IV oral submucous fibrosis and 5 male patients with oral leukoplakia with a size of 3–5 cms in diameter) from clinical and histopathological analysis. The collagen membrane used for the study is *NEÜSKIN*^*TN*^ manufactured using patented technology developed by CSIR (Council of Scientific and Industrial Research), New Delhi had dimensions of 5 × 5 cm and 0.3 mm thickness. The parameters such as pain, swelling, allergy, biodegradability of collagen membrane, degree of re-epithelisation, degree of contracture, mouth opening and wound size were assessed after the placement of collagen membrane in oral mucosal defects.

### 2.1 Pain after placement of collagen membrane

The collagen membrane was effective in relieving pain. Pain was assessed using a plain horizontal visual analog scale with a score of 0–10. A score of 0 indicated no pain, a score of 2.5 indicated mild pain, a score of 5 indicated moderate pain, a score of 7.5 indicted severe pain and a score of 10 indicated worst possible pain (Table 1).

**Table 1:** Patient data.

**S. no.**	**Age**	**Sex**	**Diagnosis**	**Pain**		**Swelling**		**Degree of re-epithelisation**			**Degree of contracture**		**Mouth opening (mm)**			**Wound size (mm)**	
				1st POD	3rd POD	1st POD	3rd POD	7th POD	14th POD	30th POD	14th POD	30th POD	Pre-OP	Intra OP	Post-OP	Initial	final
1	40	male	OSMF	25	0	0	0	poor	fair	good	good	good	41	45	44	31 × 25	30 × 24
2	40	male	OSMF	5	25	5	25	poor	fair	fair	good	good	22	45	41	35 × 31	32 × 30
3	24	female	OSMF	25	25	25	25	poor	fair	fair	fair	fair	15	40	32	50 × 25	46 × 22
4	60	male	OSMF	25	25	0	0	poor	fair	good	good	good	24	44	39	35 × 25	34 × 23
5	28	female	OSMF	25	25	25	0	poor	fair	good	good	good	17	41	34	38 × 26	34 × 24
6	58	male	Leukoplakia	5	25	0	0	poor	poor	poor	poor	poor	55	55	55	38 × 30	35 × 24
7	55	male	Leukoplakia	5	25	0	0	poor	fair	good	fair	fair	52	52	52	35 × 31	32 × 28
8	45	male	Leukoplakia	25	0	0	0	poor	fair	good	fair	fair	41	41	41	37 × 37	36 × 34
9	35	male	Leukoplakia	5	25	25	25	poor	fair	good	fair	fair	65	65	65	30 × 30	28 × 28
10	43	male	Leukoplakia	25	0	0	0	poor	fair	good	good	good	42	42	42	38 × 32	35 × 31

Abbreviations for the Table 1 OSMF – Oral submucous fibrosis, POD – Postoperative day, Pre-OP – Preoperative, Post-OP – Postoperative.

### 2.2 Swelling after placement of collagen membrane

The swelling after placement of collagen membrane was assessed using a plain horizontal visual analog scale with a score of 0–10. A score of 0 indicated no swelling, a score of 2.5 indicated mild swelling, a score of 5 indicated moderate swelling, a score of 7.5 indicted severe swelling and a score of 10 indicated worst possible swelling (Table 1).

### 2.3 Allergy after placement of collagen membrane

Systemic or local allergic reactions to collagen membrane were not seen in the 10 patients studied.

### 2.4 Biodegradability of collagen membrane

**Fig. 1:**
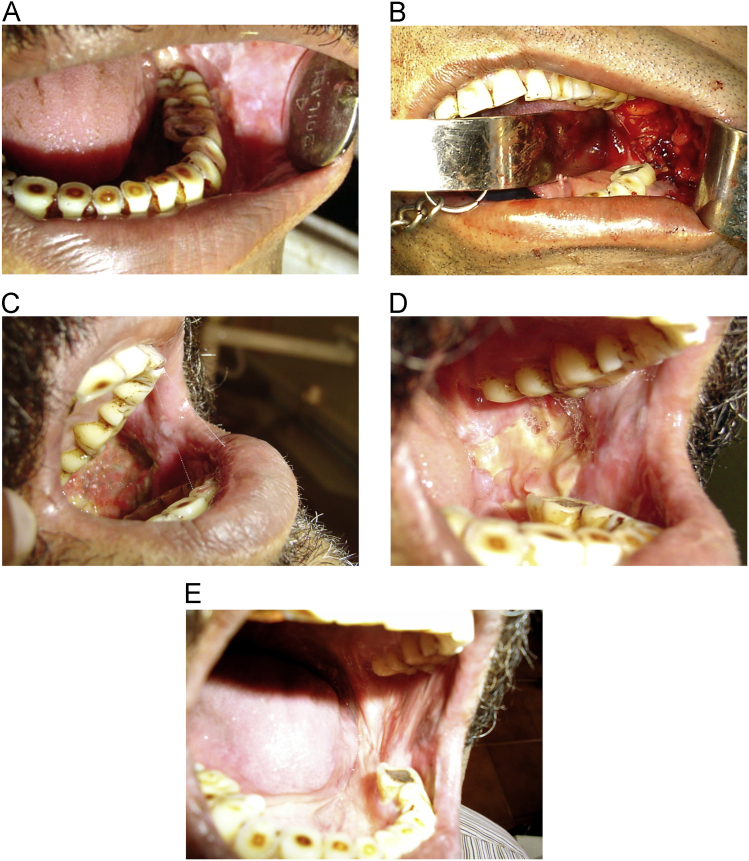
(A–E)-Clinical evaluation of oral submucous fibrosis. A: Preoperative: oral submucous fibrosis of right buccal mucosa, B: Intraoperative: collagen membrane placed over raw wound, C: Seventh postoperative day, D: Fourteenth postoperative day, E: Thirtieth postoperative day.

**Fig. 2:**
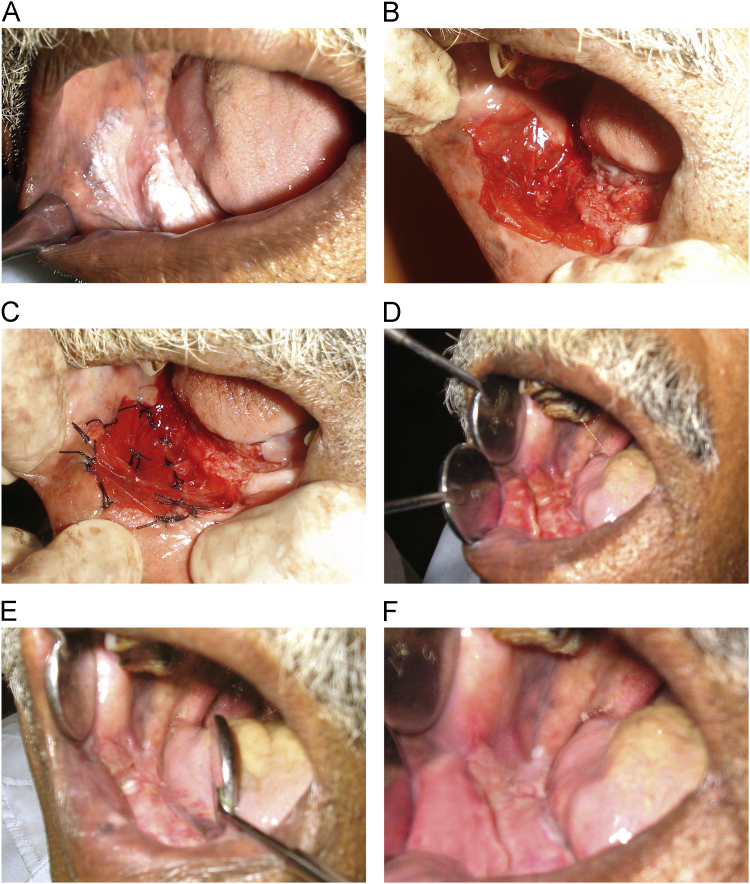
(A–F)-Clinical evaluation of leukoplakia. A: Preoperative: leukoplakia of left buccal mucosa and left alveolar mucosa, B: Intraoperative: collagen membrane placed over raw wound, C: Intraoperative: collagen membrane sutured to surrounding tissue, D: seventh postoperative day, E: Fourteenth postoperative day, F: thirtieth postoperative day.

When placed directly on the raw wound the collagen membrane underwent lysis within seven days. After 7 days most of the collagen peeled off and the remnants were removed by cutting it and by irrigating with normal saline. By the end of 7 days collagen membrane was seen to induce granulation tissue and in promoting rapid epithelialization (Figs. 1C and 2D).

### 2.5 Degree of re-epithelization after placement of collagen membrane

Degree of re-epithelization was graded as poor, fair and good based on clinical evaluation of the operative site (Table 1).

### 2.6 Degree of contracture after placement of collagen membrane

Degree of contracture was graded as poor, fair and good based on clinical evaluation of the operative site (Table 1).

### 2.7 Mouth opening after placement of collagen membrane

The postoperative mouth opening was found unaltered in cases of oral leukoplakia. In cases of oral submucous fibrosis, improvement of mouth opening ranged from 32 to 44 mm (Table 1, Table 2).

**Table 2:** Mouth opening.

**Paired samples test**				
	**Paired differences**		***Z***	***P***
	**Mean**	**Standard deviation**		
**Mouth opening preoperative to mouth opening intraoperative**	− 9.6000	11.66381	2.023	0.043
**Mouth opening preoperative to mouth opening postoperative**	− 7.1000	8.62103	2.032	0.042
**Mouth opening intraoperative to mouth opening postoperative**	2.5000	3.20590	2.023	0.043

### 2.8 Wound size

Comparison of wound size initially and at the end of 30 days revealed insignificant wound contracture, (*p* = 0.059) (Table 1, Table 3).

**Table 3:** Wound size.

**Paired samples test**				
	**Paired differences**		***t***	***p***
	**Mean**	**Standard deviation**		
**Initial wound size to wound size 30 days**	193.0000	235.13117	1.984	0.059
