# Supplementary material for: Data regarding clinical evaluation of collagen membrane in oral mucosal defects due to oral submucous fibrosis and leukoplakia
Source: Data Brief. 2018 Mar 22;18:607–12. doi: 10.1016/j.dib.2018.03.070 (PMC5996286; doi:10.1016/j.dib.2018.03.070)
Supplement: Supplementary file 1 — Supplementary material [file mmc1.doc]

**Conflict of interest**

Dear Editor

I and my co author, Dr Veeraiah Chowdary Jasthi did not have any conflict of interest in publishing the research work in the Data in Brief.

Best Regards

Dr. Thorakkal Shamim
